# Connected Bike-smart IoT-based Cycling Training Solution

**DOI:** 10.3390/s20051473

**Published:** 2020-03-07

**Authors:** George Catargiu, Eva-H. Dulf, Liviu C. Miclea

**Affiliations:** 1 Department of Automation, Faculty of Automation and Computer Science, Technical University of Cluj-Napoca, Memorandumului Str. 28, 400014 Cluj-Napoca, Romania; George.CATARGIU@student.utcluj.ro (G.C.); Liviu.Miclea@aut.utcluj.ro (L.C.M.); 2 Physiological Controls Research Center, Óbuda University, H-1034 Budapest, Hungary

**Keywords:** connected bike, smart technologies, IoT, personalized training, embedded, back-end, front-end, Android, modules, MQTT, monitoring

## Abstract

The Connected Bike project combines several technologies, both hardware and software, to provide cycling enthusiasts with a modern alternative solution for training. Therefore, a trainer can monitor online through a Web Application some of the important parameters for training, more specifically the speed, cadence and power generated by the cyclist. Also, the trainer can see at every moment where the rider is with the aid of a GPS module. The system is built out of both hardware and software components. The hardware is in charge of collecting, scaling, converting and sending data from sensors. On the software side, there is the server, which consists of the Back-End and the MQTT (Message Queues Telemetry Transport) Broker, as well as the Front-End of the Web Application that displays and manages data as well as collaboration between cyclists and trainers. Finally, there is the Android Application that acts like a remote command for the hardware module on the bike, giving the rider control over how and when the ride is monitored.

## 1. Introduction

As times change, technology evolves and people migrate from fieldwork to deskwork, a new health threat arises: the sedentary lifestyle. To fight this phenomenon, fitness seems like a handy solution, especially for the advantage of having a personal trainer that can monitor the activity and offer guidance throughout the training. The electric bicycle is a trendy solution, as it is presented in [1]. However, most cyclists either amateur or professionals do not have this opportunity, making it harder to improve and reach specific goals. This paper proposes a technical solution meant to overcome this issue, by using sensors, microcontrollers and microcomputers to read, send, save, and display data. Therefore, any trainer can monitor relevant cycling data gathered from the bike in real time.

Data collection from sensors is widely used today. However, all the data are focused on sharing system development [2,3,4], sensor network implementation [5,6], safety [7], smart city development [8,9] or intelligent control implementation [10,11,12]. A series of great results are published considering the positioning method of the sensor system, structural health monitoring, and intelligent vehicle [13,14,15,16,17]. No research focusing on IoT (Internet of Thinks) and smart bike fusion for training has been published.

Nowadays, in cycling, the most common training method is by sheet. This sheet can be custom made by a trainer for one or more riders and holds a training plan by days, weeks and even season periods. However, this old and rigid method has a higher chance of failing to reach its purpose.

The Connected Bike proposed in this paper retrieves and transmits over internet the speed, cadence and cyclist power output both to a server-hosted database and to a web application, from where a trainer can monitor, evaluate and notify possible errors made by the cyclist during the ride. Furthermore, by persisting data to a server, both the cyclist and the trainer can return over past rides and discuss about them. The rider also has the possibility to control the system by starting, pausing or ending the ride with the help of a mobile application and internet connection.

All the technologies used in the presented Connected Bike project serve the following purposes:

- Collecting data from sensors
- Computing the speed, pedaling cadence and instant power output of the cyclist from collected data
- Real time GPS tracking of the bicycle
- Aggregating and sending data to the web interface for online monitoring
- Persisting training data to a database for later visualization
- Allowing user to control the monitoring system through a mobile Android app
- Implementing security levels to protect users’ data

The idea of real time bicycle tracking has been around for a while, and Chris Kiefer and Frauke Behrendt tackled this in [18] with a smart e-bike monitoring system (SEMS). They use custom hardware and a mobile phone with internet connection as an access point to monitor data like the bike’s current location and the motor’s level of assistance. This project is aimed towards e-bicycle fleets, focusing on scalability and ease of use, thus leaving aside acquisition of training relevant data like speed or cadence. A significant drawback for SEMS is that the battery powering the system discharges quite fast because of the hardware and connected mobile phone.

Neil Savage describes in [19] a bicycle data-monitoring concept named Copenhagen Wheel, by replacing the rear wheel with an intelligent one. This wheel has a modified hub, which encapsulates in addition to a standard one, an electric motor, and an ensemble of electronic components, both powered by a battery. The system is capable of changing gears, adapting the motor’s speed and monitoring the torque, all while gathering pollution, humidity, temperature data, and sending it to a server through a mobile phone connected to the internet.

In terms of gathering data, which is a key concept of this research, Maurizio Di Paolo Emilio presented in [20] the main concepts involved in the process. Therefore, he described a data acquisition system as a mean of collecting information about a physical phenomenon through sensors and other electronic components. The sensors can be both digital (1/0, ON/OFF, etc.) and analog, the latter being harder to quantify and needs additional sampling and conversion circuitry. Any data acquisition system must have a computer or microcontroller as a core in charge of processing data and a mean of transportation, storage and display for this data.

In [21], Sarah Al-Mutlaq briefly presented the working principle of a load cell. These sensors translate the load or force acting on it into an electric signal and can be of three types:

1. Hydraulic load cells—determines the applied force by measuring the displacement of a piston in a cylinder and the change of a diaphragm, which modifies the pressure inside the Bourdon tube
2. Pneumatic load cells—measures the force by determining the air pressure applied to one end of the diaphragm, which then leaves the cell through a nozzle on the other end.
3. Strain gauge load cells—mechanical elements, without moving parts that measure the force through the deformation of one or more strain gauges.

All these ideas are embedded in the present paper, leading to an easy-to-use smart bike for personalized training. The novelty of the paper consists in the design and implementation of this smart connected bike, using the interaction of future-oriented mobility and state-of-art data technology, but cheap and accessible elements. All steps are described in detail to be easy to reproduce by the reader. The presented solution has the potential to disrupt existing training solutions.

The paper is structured in three parts. After this short introductory part, Section 2 reveals the used materials and methods, divided in 12 subsections, for each discussed module. Section 3 presents the obtained results. The work ends with concluding remarks.

## 2. Materials and Methods

The interface for monitoring the cyclist’s activity must have an increased portability and must be easy to access and interact. To meet these criteria, a web application was developed. This can be accessed by trainers, as well as by cyclists, having pages and functionalities tailored for the specific user type.

In the web application, a trainer has access to a list of the cyclists to whom they have connected, can select a rider for real time monitoring, or analyze any of the previous rides. They can also stop the collaboration with any rider or establish a new collaboration with another. Last, but not least, the user has access to a page from where they can view and modify their personal account’s data. The cyclist user cannot see their performance in real time, having only access to the previous rides. However, they have the possibility to control the monitoring by creating a new ride, pausing or ending the current one, through the help of a mobile phone application that acts as a remote for the module on the bicycle.

From the hardware point of view, the project consists of three modules: a microcontroller module, a microcomputer module and a mixed one. The first one is in charge of reading data from the load cell, measuring the force applied to the pedal and sending the data to the second module through a wireless channel.

The second module consists of a microcontroller, in charge of reading and processing data both from sensors and from the first module and a microcomputer that receives this data, converts it into a standardized message format, and sends it to the server via internet. It also retrieves the current location in terms of latitude and longitude with the help of a GPS module.

Last, but not least, the third module is an always-on microcomputer which hosts the back-end of the web application and the MQTT broker. Two rechargeable batteries power the first two modules, and the mixed module requires internet access that is provided either by a mobile hotspot or by an internet USB Stick.

Taking into account the multiple IoT specific architectures, described by P.P. Ray in [22] and presented shortly in the previous section, the most suitable one for this research proved to be a hybrid architecture, including the sensors, microcontrollers and the web and mobile applications.

**Figure 1:**
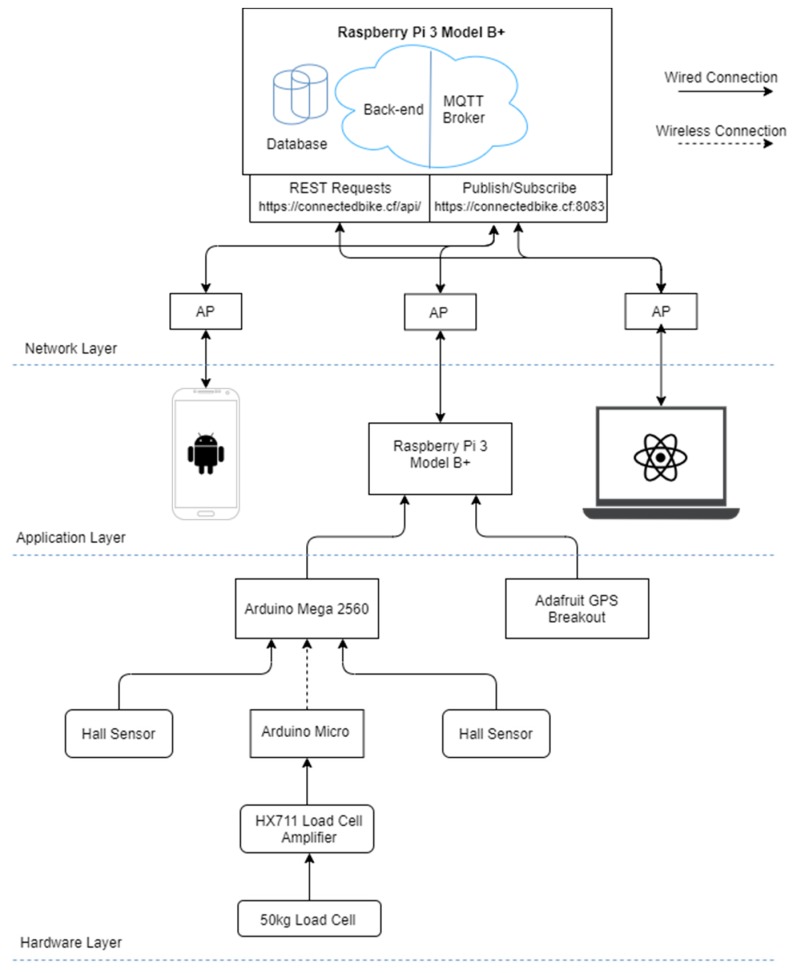
Connected Bike system architecture.

As seen in Figure 1, the entire system is structured on three levels: the hardware level, the application level and the network level.

The hardware level consists of two modules that both have an Arduino microcontroller with separate power banks and have wireless communication between each other. The first module is based on an Arduino Micro, having connected an amplification module HX711 and a load cell. This module reads data from the load cell and sends it to the other module via infrared communication. The second module has an Arduino Mega with two Hall Effect sensors, used to determine the speed and cadence. The microcontroller performs data manipulation, afterwards converting it to byte array and sending it to the Raspberry Pi through USB communication. Here, the data is converted again from binary format and is aggregated with latitude and longitude readings from the GPS module. The final data format is a JavaScript Object Notation (JSON) that is periodically published to the server.

The application level includes three main components in charge of the system’s business logic, establishing the way the application is handled and how data is stored and displayed. The first component is an Android App that is used to command the Raspberry Pi located on the bicycle, making it easy to manage the ride monitoring. The second component is the Raspberry Pi module placed on the bicycle that implements the system’s logic in the form of a state machine, deciding based on the commands from the Android App if it should start reading, managing and sending data. Finally, the third component of this layer is the web application, where users can register, login and view different rides’ data, or monitor a ride in real time.

The network level is made out of the actual communication between modules through internet. All the IoT-specific communication in this system is based on the MQTT, each component being connected through an Access Point as clients, except of a Raspberry Pi device, which is the actual server, hosting both the MQTT broker and the HTTP RESTful API server for the web application. There is also HTTP protocol used to establish communication between the front-end and the back-end for the web application.

**Figure 2:**
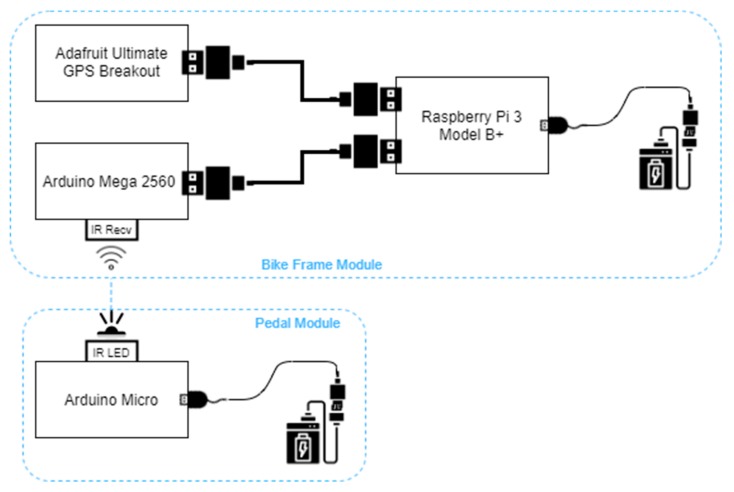
The hardware scheme.

Seen as a black box, the entire hardware scheme is described in Figure 2, making it easy to observe the way modules communicate with each other. The module blocks are highlighted with blue dotted lines. The communication between the Arduino Mega, Raspberry Pi and the GPS module is realized via USB (for the GPS module, an UART to USB converter was attached). Furthermore, because there are two rotating joints from the pedal to the bicycle’s frame, making it harder to use wires, the infrared transmission medium was chosen for its simplicity and robustness. The downside of this approach was the addition of another microcontroller and an extra battery to power it. 

### 2.1. The Pedal Module

**Figure 3:**
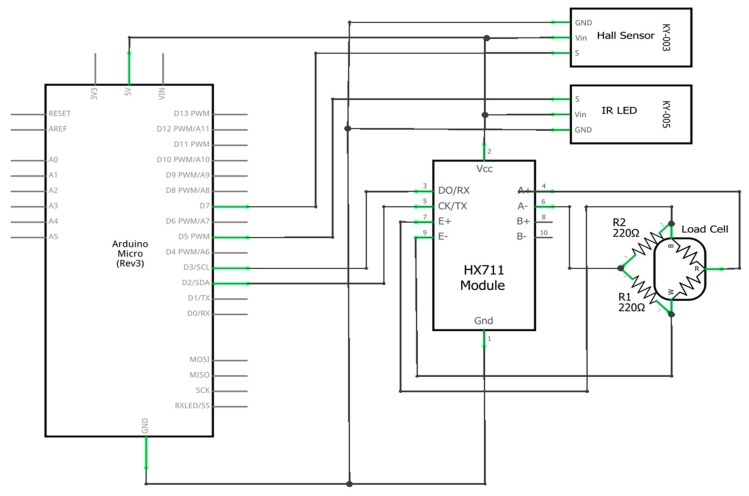
Pedal module wiring diagram.

The main functionality of this module is reading the force applied to the pedal by a cyclist. This is possible thanks to a load cell, an amplifying driver, a microcontroller, and a Hall Effect sensor. The data is sent to the frame module with an infrared LED connected to the microcontroller. A rechargeable portable battery powers the entire module. The wiring diagram is shown in Figure 3.

The microcontroller implements all the logic in this module, being responsible for reading, processing and sending data with a sample frequency of 80 Hz. Because of the fitting space and power limitations, an Arduino Micro was chosen for this module. The communication between the Arduino and the load cell driver is based on I2C (Inter-Integrated Circuit) using the pins 2 and 3 configured as serial data and serial clock. The Hall Effect sensor was connected to General-Purpose Input/Output (GPIO) pin 7, configured as an external interrupt pin and for the infrared LED, the fifth GPIO pin was used.

For this project, a rectangular load cell from SparkFun was used. Thanks to its rectangular form and small dimensions, it fitted directly on the pedal, receiving the full foot force from the cyclist. This load’s range is between 0 and 50 kgf and measures with ± 0.5%RO accuracy.

Another particularity of this sensor, in contrast to its alternatives, is the number of wires. Most of the load cells provide four wires, making it easy to use one standalone sensor because it contains a full Wheatstone bridge made out of strain gauges. However, this particular load cell uses three wires and implements half of the bridge from two strain gauges of 1000 Ω each, connected in series.

The HX711 driver was developed for general interfacing between a microcontroller and a load cell, working with a voltage supply of 2.7 to 5 V and has a very simple working principle. Two pins are sending an excitation signal to two sides of the Wheatstone bridge (from the load cell) and two other pins are reading the voltage difference between the other two sides of the bridge. In free mode when no force is applied, the bridge is balanced, thus the difference is zero. When a force is applied onto the cell, the bridge becomes unbalanced and the voltage difference increases. The HX711 reads this difference in mV, amplifies it and maps it to digital values to be sent to the microcontroller.

In order to make this module work with only one three wire load cell, the bridge had to be completed with two 220 Ω static resistors, allowing the driver to read the voltage change from the sensor.

In order for the data to be received from the HX711 driver and processed by the Arduino Micro to reach the frame module, an infrared 5 mm LED was used. Because the pedal is continuously rotating and the pedal module is functioning as a standalone one, a Hall effect sensor was used to determine the position of the pedal arm relative to the bicycle’s frame so the Arduino Micro gets notified when the pedal reaches a full circle and passes the magnet placed on the frame.

### 2.2. Bike Frame Module

**Figure 4:**
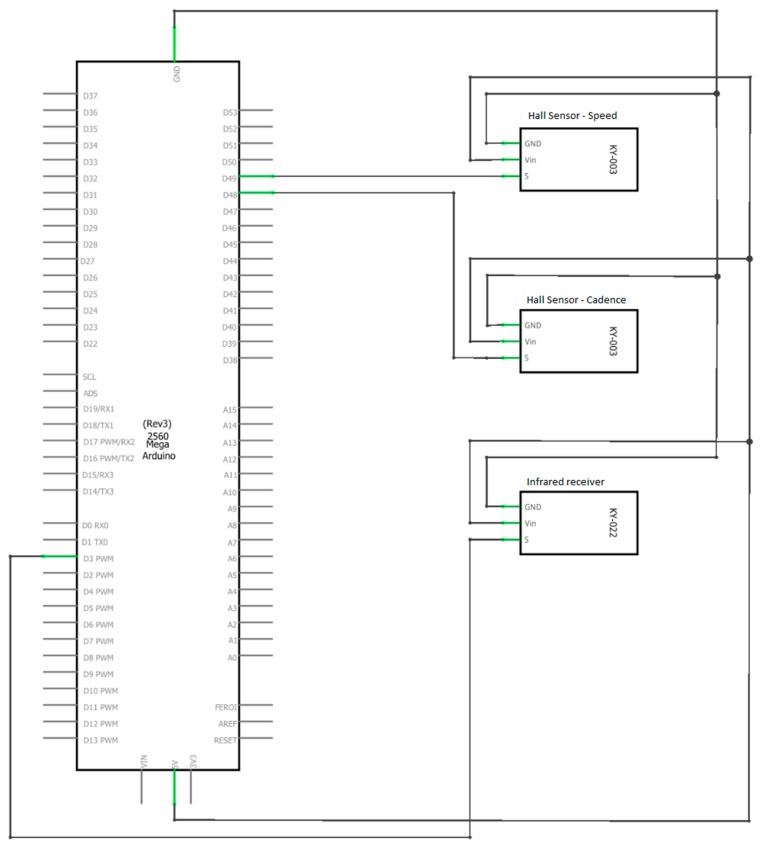
Bike frame module wiring diagram.

This module is used to determine the speed, cadence and rider’s power during the ride activity. All this data is serialized and sent via USB to the Raspberry Pi, which acts as a master of the entire system, but also as an internet gateway. Being also a standalone module, it is powered by a rechargeable battery. The wiring diagram is presented in Figure 4.

For data processing, an Arduino Mega 2560 was used, which communicates with the Raspberry Pi via USB. The 16 MHz processing frequency of the microcontroller allows implementation of multiple tasks in a Real Time Operating System (RTOS). The necessity of using an RTOS occurred from the need to read and compute sensor values with higher precision and data transmission.

To monitor the rider’s position continuously, a GPS module from Adafruit was used. This device has a sensitivity of −165 dB, 10 readings per second, 66 channels, and the possibility to receive signals from 22 satellites with its own integrated antenna. The latitude and longitude are sent via a FTDI USB-TTL converter to the Raspberry Pi.

### 2.3. Software

This project was built using multiple programming languages and technologies, addressing different parts and modules.

**Figure 5:**
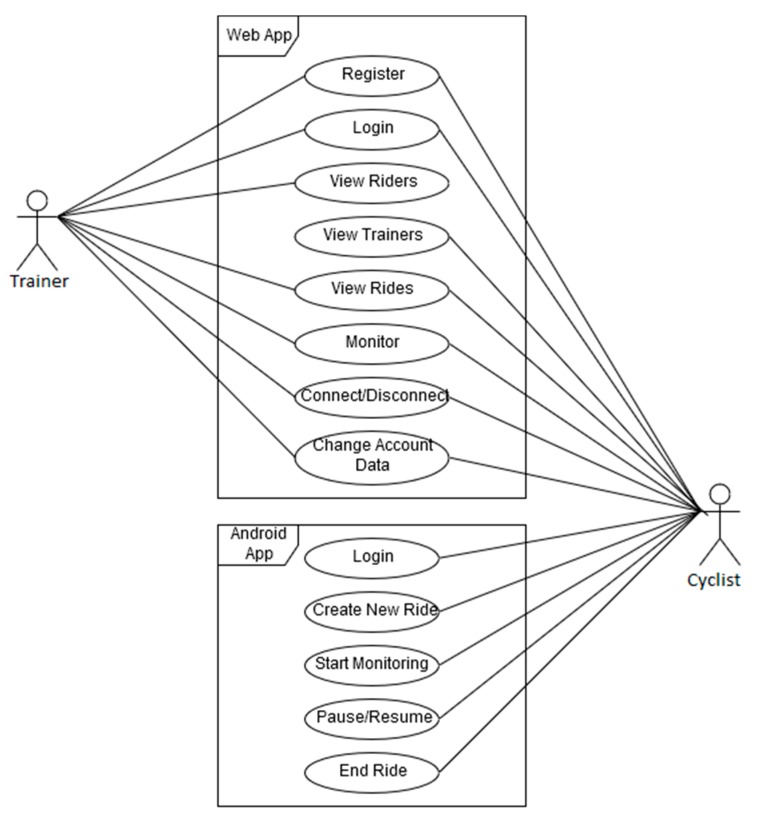
Connected bike use cases.

The users can interact with the system using two applications: An Android app and a web app. Riders can only use the first application while both riders and trainers can use the second app. The user’s use cases diagram is presented in Figure 5.

In the following sections, the software implementation is explained, alongside the used technologies for each module.

### 2.4. The Pedal Software Module

The main component for this module is an Arduino Micro, detailed in the previous hardware chapter. The open source libraries used to implement the software for this microcontroller are HX711.h and IRemote.h. The first one allows interfacing with the HX711 load cell driver while the second one was used for sending encoded message with the infrared LED. The setup function from the Arduino sketch initializes the pin connected to the IR LED and attaches a rising edge interrupt as well as an interrupt service routine to be dispatched on each event. Following this, a HX711 driver initialization and calibration sequence is executed.

Once the setup is complete, the loop function is called, reading the sensors in an infinite loop. The load cell reading is performed by the get_units function from the library mentioned above. The returned value of this function is converted from lbs. to kg and scaled by 10 for a single decimal precision as seen in Equation (1).

(1) val[kg]=(val[lbs]·0.453592)·0.4

The readings are stored in a three values circular buffer holding the highest values read during a complete pedal cycle. Once the pedal reaches a full rotation and comes next to the magnet placed on the frame, the Hall Effect sensor triggers an interrupt on pin 7, therefore triggering the corresponding interrupt routine. This function computes the average between the values from the buffer, sets the transmission flag and clears the buffer. In the loop function, on each iteration, the transmission flag is checked and if it is set, then the previously computed average is serialized and sent via USB to the Raspberry Pi.

### 2.5. The Frame Module

**Figure 6:**
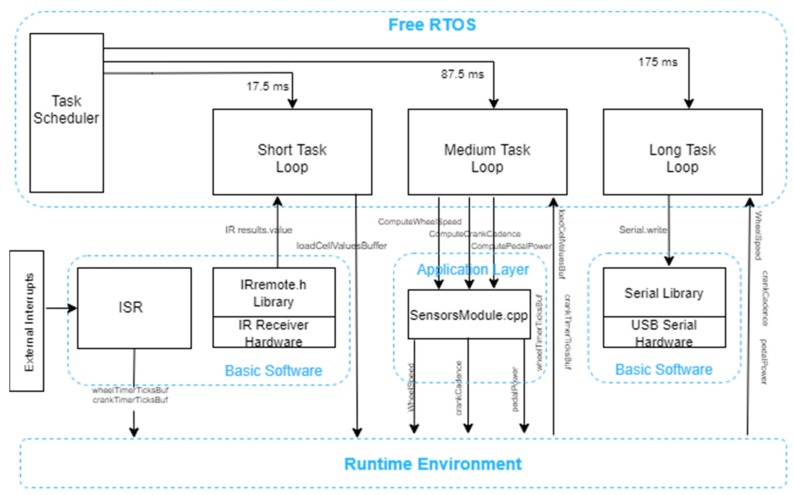
Software architecture.

The code for the Arduino Mega 2560 in this module is structured in multiple files with the .cpp and .h extensions. The software architecture for the frame module is presented in Figure 6.

The code structure and the data manipulation follow a custom-made structure, following guides and patterns from an automotive. Thus, as seen in the above figure, the data transmission and the functionalities are decoupled, data travelling from one component to another through a virtual medium named the Runtime Environment, implementing an AUTOSAR-like (Automotive Open System Architecture) architecture, adapted to the application’s context and simplified. The code is structured in four abstraction layers:

- Basic Software—this level is dedicated to hardware interaction between the microcontroller and its connected peripherals. Here can be found the libraries used to interface the sensors and drivers and also the interrupt service routines
- Runtime Environment—as the name suggests, this is a software medium, which only exists at runtime. This holds global variables and value buffers used to update and send data between abstraction layers creating the actual decoupling between layers
- Application Layer—represents the business logic of the program and includes all the functions for computing speed, cadence and pedal power. The raw sensor data is received via runtime environment from the hardware layer, and the computed values are returned back to the RTE from where will they be taken by the transmission function, sending them over USB to the Raspberry Pi
- RTOS (Real-Time Operating System)—is the component in charge of the entire program’s execution flow, triggering three periodic tasks based on the priority and task periodicity
- When powering up the microcontroller, the infrared receiver library is instantiated and the setup function is called. Here the following functions are triggered:
- initPinout—initialize pins 48 and 49 for the two Hall effect sensors
- set the pins as digital inputs
- activate the pull-up resistor
- initTimers—timers 4 and 5
- reset the TCCRnA (n = 4,5) registers
- set the prescaler to 1024
- set the capture and overflow interrupt
- reset the interrupt flag register
- irrecv.enableIRIn—activate the infrared receiver
- xTaskCreate—create three tasks for the RTOS and set the execution priority for each
- sei—activate global interrupts
- vTaskStartScheduler—start scheduling component from the RTOS in charge of periodically calling the tasks based on the priority

The initPinout and initTimers macros initialize the pins and timers used by the program and are placed in a separate header file called ucActuatorInit__hh.h using the *#define* directive.

The IRrecv library for the infrared receiver is instantiated at the beginning and is activated by the enableIRIn function. After creating an instance, the object reads in the background the data from the KY-022 module and updates the global results variable, of type decode_results.

**Figure 7:**
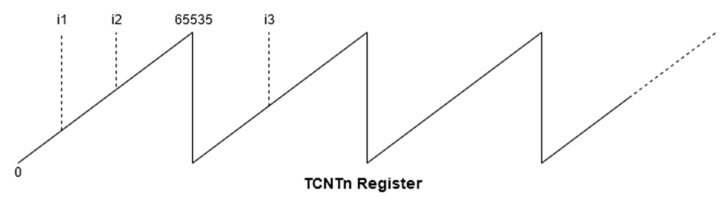
Timer’s working principle.

In order to compute the speed and cadence, the timers four and five are used. When initialized, the timers are configured to use in normal mode and the capture mode is activated. This means that each timer will increment the TCNTn (Timer Counter Register n = 4, 5) starting from 0 to the maximum value of 65535, and once it reaches this value, the register is reset. Each timer has an allocated pin for the capture mode (pins 48 and 49). Thus, by enabling this mode, the pins’ functionality is also changed, allowing them to trigger interrupts on events such as a Hall Effect sensor’s impulse, capturing the TCNT’s current value in a separate register ICR (Interrupt Capture Register), and an interrupt service routine is dispatched. The timer’s working principle is presented in Figure 7, presenting a normal mode of operation and three interrupts on Interrupt Capture Pin.

Both the timer 4 and timer 5 routines implement the same operations. First, the tick count is read, then using the tick count from the last interrupt, the elapsed ticks between two consecutive interrupts are determined, using Equation (2), and the result is stored in a circular 10 values buffer from the runtime environment.

(2) $$t_{timer}=i_{2}\left(k\right)-i_{1}\left(k-1\right)$$

where *k* refers to current ticks and (*k*−1) to previous ticks.

In the case where the current timer value reading is lower than the previous one, the tick count will be determined with Equation (3).

(3) $$t_{\mathrm{timer}}=65535-i_{2}\left(k-1\right)+i_{3}\left(k\right)$$

The SensorsModule.cpp file holds the functions to calculate the speed, cadence and power. These functions take the value buffers as parameters and compute the average for the entire set. The value buffers from RTE are defined as volatile ensuring atomic access and data consistency. The speed computing function determines the bicycle’s speed implementing as in Equation (4).

(4) $$v_{wheel}=\frac{C_{\mathrm{wheel}}}{t}\cdot 3.6\cdot 10$$

where *v_wheel_* represents the actual wheel speed, *C_wheel_* is the wheel circumference in [mm], and t is the time necessary for rotation in [ms]. The value 3.6 is the 1 m/s converted in 3.6 km/h, and the value 10 is the scaling preventing from working with float values.

Similar to the previous function, the cadence calculus follows Equation (5).

(5) $$\mathrm{Cadence}=\frac{\mathrm{time}}{\frac{\left(\mathrm{timerTicksAvg}\cdot 64\right)}{1000}}$$

where time is measured in [ms], while timerTicksAvg in [μs].

The power computing function, in contrast to the previous ones presented above, receives both the 10 values buffer holding the load cell readings and the current cadence, necessary to determine the angular speed used in the final formula. The instantaneous power is determined with Equation (6).

(6) $$P_{\mathrm{pedal}}=F_{\mathrm{pedal}}\cdot l\cdot \Omega$$

where *P_pedal_* represents the pedal power, *P_pedal_* the pedal force, *l* is the crank leg length measured in [cm], and *Ω* is the angular speed.

**Figure 8:**
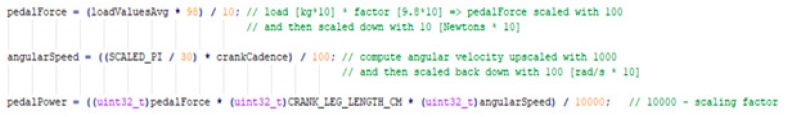
C code implementation of power computing.

At the microcontroller side, the implementation of the power computation is presented in Figure 8.

The most important software part implemented on the Arduino Mega is the real time operating system. It completely replaces the implicit function loop with periodically executed tasks. At the core of this framework stands the task scheduler. This component dispatches tasks based on their period and priority.

For this project, the FreeRTOS library was used, developed as open source under the MIT license [23]. In order for the library to work on most microcontrollers available on the market, the timer used to determine when a task should be dispatched is the watchdog timer. In addition, because some of the microcontrollers including Arduino only have a single priority level, the library implemented an abstraction layer exposing four virtual priority levels.

In the ucActuator.ino file, inside the setup function, three tasks are created with different periods and priorities, using the xTaskCreate from the FreeRTOS library as follows:

- shortTask
  - 17.5 ms execution period
  - Highest priority
  - Continuously interrogates the infrared receiver and saves the values to the RTE buffer and performs the same for the pedal load cell
  - Operations implemented in this task have the shortest execution time
- mediumTask
  - 87.5 ms execution period
  - Medium priority
  - Calls the functions that compute the speed, cadence and power and returns the results back to the RTE
  - Operations performed in this task have a medium execution time
- longTask
  - 175 ms execution period
  - Lowest priority
  - Checks if the timers used for speed and cadence overflowed more than twice, meaning the movement stopped and sets the corresponding values to 0
  - Calls the function that transmits the serialized data via USB to the Raspberry Pi
  - Data serialization to byte array and data transmission take the longest time

**Figure 9:**
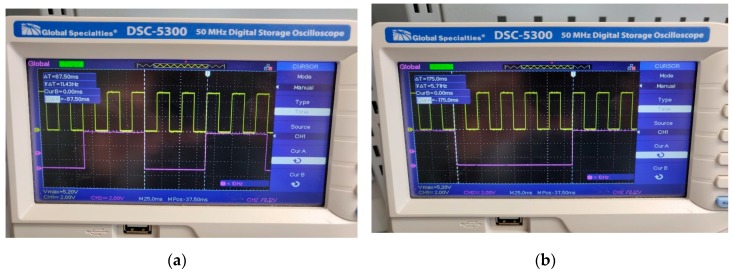
(**a**) Medium (purple) and short task (yellow); (**b**) Long (purple) and short task (yellow).

Due to the software priorities, the tasks are executed preemptively. Thus, the medium task is interrupted five times by the shortest task, while the longest task is interrupted 10 times by the short task and twice by the medium task. This allows for a so-called parallel execution on a single core processor given also the reasonable 16 MHz processor frequency of the Arduino Mega. A visual representation of tasks can be observed in Figure 9.

To facilitate code maintenance, a configuration header was created named ucActuator_kh.h containing pin names and constant values used throughout code. In the event a pin or a parameter shall be changed, all the modifications can be performed into a single file.

### 2.6. The Raspberry Pi Frame Module

The Raspberry Pi from the frame module is in charge of processing data from Arduino Mega, structuring it into messages and sending them over the internet to the server and front-end for storing and visualization.

The software for the Raspberry Pi application is written in Python, and the communication wireless communication is performed through the MQTT (Message Queueing Telemetry Transport) protocol. As presented on the official web page [24], this protocol is a simple way of sending data between nodes as clients via TCP or Web sockets, used mainly in Internet of Things. The two main entities that are at the core of this protocol are the client and the broker. The latter manages the communication between clients that can publish messages to a topic or subscribe to a topic to consume messages. The Python library that was used to work with this protocol is Paho, which was developed as open source by the Eclipse foundation.

The code was structured by functionalities, in multiple files with the .py extension, also called Python modules. The entry point of the application is the piBrain.py module where two methods are called from the beginning. The first one blocks the program’s execution waiting for an internet connection, returning when one is established. The next method is the main one, where the configuration file appconfig.cfg is parsed and the constants are retrieved. In addition, this method instantiates the MqttRemoteThread class and starts the thread.

**Figure 10:**
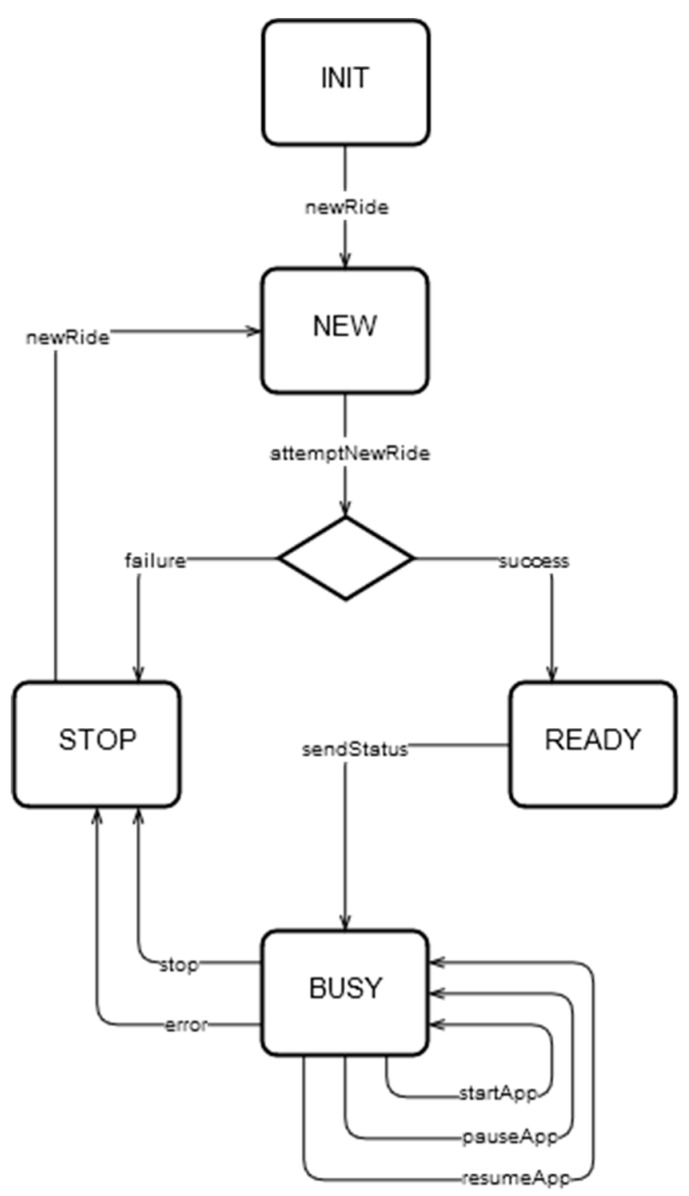
State machine of Raspberry Pi application.

The MqttRemote.py module contains the MqttRemoteThread class, which extends the Threading class, allowing it to run in a separate thread. The class implements a state machine, listens to commands coming from the Android App through MQTT, and based on them changes the state of the application. Therefore, the cyclist can start, pause or stop the ride using their mobile phone, the thread modifying the entire application’s state. A visual representation of the state machine can be seen in Figure 10.

When instantiating the MqttRemoteThread, a new MQTT client is also created that subscribes to the command topics that are sent from the Android App. Paho allows for attaching callback methods to events triggered on-message. The first method of such a kind is named on_connect and is dispatched right after a new connection is established between the client and the broker. Here, the client subscribes to the specific topic. The second callback method is on_message and it is called each time a new message is published to the topic the client is subscribed to. The library also provides a loop_start method that creates a new non-blocking thread that will get messages by polling on the subscribed topics, calling the on_message method once a new message is detected. In this application, the commands that the Android App can send are:

- “NEW”—creates a new ride;
- “START”—starts the current ride;
- “PAUSE” —pauses the current ride,
- “RESUME”—resumes the current ride,
- “STOP”—end the current ride

A new ride is created by the attemptNewRide method, which performs five tries as POST requests to the server. If the server returns 201 success codes together with the newly created ride’s primary key, then the program saves this id that is afterwards used to save the ride data to the corresponding ride id. If the server fails to create a new ride, then the application changes its state to “STOP”, issuing an event through MQTT back to the Android App to inform the user about the failure.

The run method of the MqttRemoteThread class runs in an infinite loop and consists of if statements that check if a new command was received, changing the application’s state accordingly. Once in a new state, the application will perform the required operation and then enter a listening state, ready to accept further commands. This thread is also responsible of sending information back to the Android App to inform the user about the current application’s state.

When a user starts the application and sends the “START” command, the startApp method is called from the main thread, which starts three new threads and a thread safe queue object for passing data between them: serialReadThread, gpsPollingThread, mqttPublisherThread.

The SerialReadThread is implemented in the SerialReader.py module, which extends the Threading class and serves the purpose of reading and decoding the data received on the USB bus. On each loop iteration, if the application’s state is “RUNNING”, the serReader method is called to read six bytes from the input buffer. The raw data is converted into the actual values for speed, cadence and power, which in turn are formatted into a dictionary object that is added to the thread safe queue from where it can be read by other threads.

Inside the GpsPolling.py module there is the GpsPollingThread that is also a separate execution thread where values for latitude and longitude are read using the gps library for Python. The values are updated periodically and stored into a dictionary object, being available through the getLocation method.

**Figure 11:**
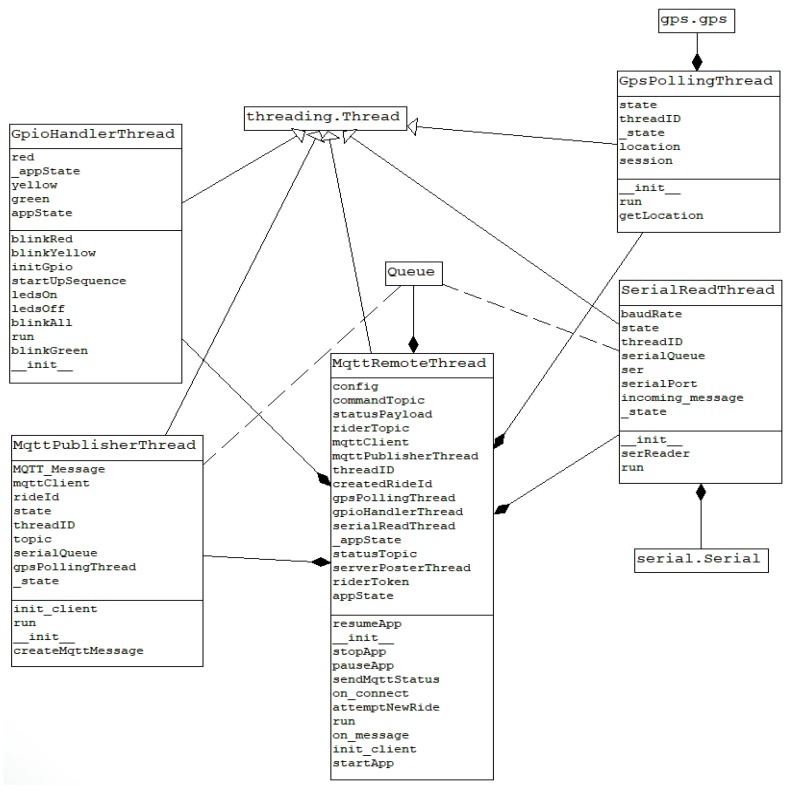
UML classes diagram.

Last but not least, the MqttPublisherThread from the MqttPublisher.py module is the execution thread where the final message is created and is sent over MQTT. The speed, cadence and power values as well as the latitude, longitude and the current date-time are retrieved from the thread safe queue object and put together in a JSON message. This message is sent to the cyclist-dependent topic. The way this application’s classes interact is presented in Figure 11, which contains the UML classes diagram in Pynthon, used in Raspberry Pi.

In order to start the application at boot time, a Linux shell script was created called raspmodule.sh. Here, the Python App’s main module, piBrain.py, is started using its own virtual environment, using the local interpreter and libraries. This shell script is triggered when the Raspberry Pi boots thanks the Cron utility available also on Debian that reads a crontab file where the script was added.

### 2.7. Android Application

The Android Application has been designed to provide a user-friendly interface and usage. The sole purpose of this application is to inform the user about the state of the bicycle module and to allow them to interact with the system using the phone as a remote control to create a new ride, to stop or resume the current one.

In terms of technical implementation, this app includes two main activities, two classes and one interface. The first activity consists of a TextView that displays in the form of a label the name of the application “Connected Bike Trainer”, two EditText elements that allow the user to enter the username and password. It should be noted that in this application, a user could only access with an account that was previously created in the web application. Thus, the last element is a login-only button that triggers the user authentication and if it proves successful, it creates the next activity.

The second activity consists of four buttons and three graphical elements of type TextView. Here, the user can interact with the application, can create a new ride, start, pause, resume, or end the current one.

Of the two classes, MqttHandler is responsible for sending commands and receiving status messages from the Raspberry Pi, via the MQTT communication protocol, while the Service class is responsible for communicating with the server via HTTP protocol.

When a user accesses the application, the first activity to be started is the Login one. Attached to the login button in this activity, is a method that will be called when the button is pressed. This method, attemptLogin, performs the necessary operations to validate the account data end and prevents sending erroneous data to the server such as blank credentials, or special characters. If the validation passes, the UserLoginTask class is instantiated and the execute method is called. This class, representing an asynchronous task, extends the AsyncTask class and contains four methods. The onPreExecute method is automatically called first and it makes the loading component available on the phone’s screen. Next, the doInBackground method is called, which has the role of logging the user into the application asynchronously. This is the main component of this task, executing the instructions in a separate thread without blocking the main thread or the graphical user interface. Here, the RequestLogin method is called from the Services class, sending the username, password and an instance of the RestCallback as parameters. onSuccess, onFailure and onNotRider are the three interface methods that are asynchronously called based on the server’s response. If the login I is successful, the current activity is replaced by the RiderActivity, which is the core of this application.

The Services class is utilized just by the login activity, implementing the RequestLogin and RequestUserData methods. The first one creates a JSON object with the user’s credentials and makes a POST request to the server. The returned response is valid and successful if it has the “token” field, representing the authorization key. Moreover, if the logged-in user is a cyclist, the onSuccess method is called, otherwise the onNotRider is dispatched, displaying a message to inform the user that only cyclists can use this Android Application to control their rides. The second method performs a GET request to the server, using the token received from the previous request and the response is valid if it contains the “topic” field. The synchronization between the asynchronous tasks and the execution threads is done with a CountDownLatch instance that has also been configured with a 5 s timeout to prevent blocking connections.

**Figure 12:**
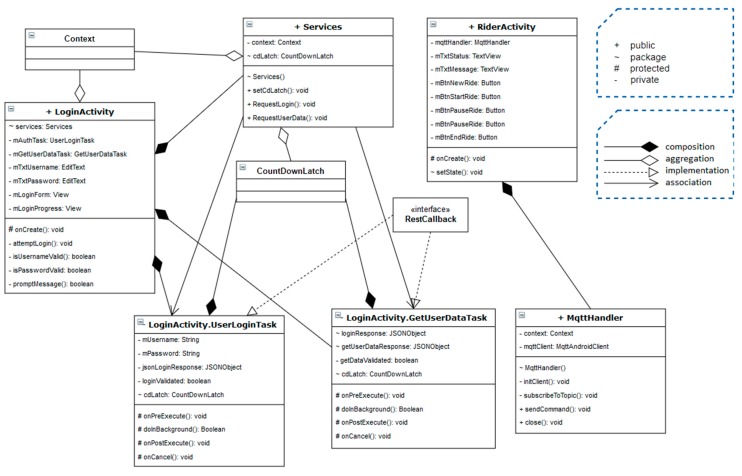
UML class diagram in Android application.

The methods by which the user can interact with the bicycle system are implemented in RiderActivity. The four buttons available to the user have those methods attached in order to send messages and change the state of the frame module application, running on the Raspberry Pi. This is possible by calling the sendCommand method implemented in the MqttHandler class. Here, the command is converted to JSON and sent on the dedicated command topic to the frame module. Also, being a two-way communication between the Android App and the Raspberry Pi on the bicycle, in order to receive and display information about the state of the frame module, when the eclipse.paho library is initialized, event handlers are attached for events such as lost connection or successful message delivery. When a new message is received from the frame module, the messageArrived is called, retrieving it and sending it for displaying in the RiderActivity to inform the cyclist regarding the application’s state. The MQTT connection to the broker is established in the constructor of the MqttHandler class. For a better understanding of the Android Application, a UML class diagram was developed, shown in Figure 12.

### 2.8. The Server

As most IoT systems, the Connected Bike project also has a server, which in this case consists of two main parts. The first part is the MQTT broker, which is necessary for the intermediation of messages between clients, while the second part is the Back-End for the web application, having both the role of persisting data transmitted from the bicycle and providing functionality to the Front-End.

Being a vital component in the operation of the entire system, the server must be switched on and have an uninterrupted Internet connection. The optimal solution was to use a Raspberry Pi 3 Model B+ to host the server for this project, being both low-cost and reliable. It is always powered on and connected to the internet, serving both the Back-End and the MQTT broker. In order to be accessible to the other modules, port forwarding rules were set on the router to which the Raspberry Pi is connected and the device received a static IP in the Local Area Network. Therefore, the other components of the system are able to access the services available on the server using the public IP of the router and the specified port. Next, are presented the two components that run on the server explaining them in more detail.

### 2.9. Back-End

The Back-End component is the one that directly accesses the database and implements the logic behind the web interface so that the data requested in the application is formatted and sent to the requester. In this project, the Back-End serves both the Front-End of the application and the Android Application. The communication protocol is HTTP, currently being one of the most used data transmission protocols over the internet.

The programming language in which the Back-End part was implemented is Python 3.6, using the open-source flask and flask-restful libraries. The design pattern used is Model-View-Controller (MVC), but only the Model and Controller are implemented on the Back-End side, because the Front-End is in charge of the View. In the development of the Back-End, the steps in the tutorial [25] have been followed and adapted.

**Figure 13:**
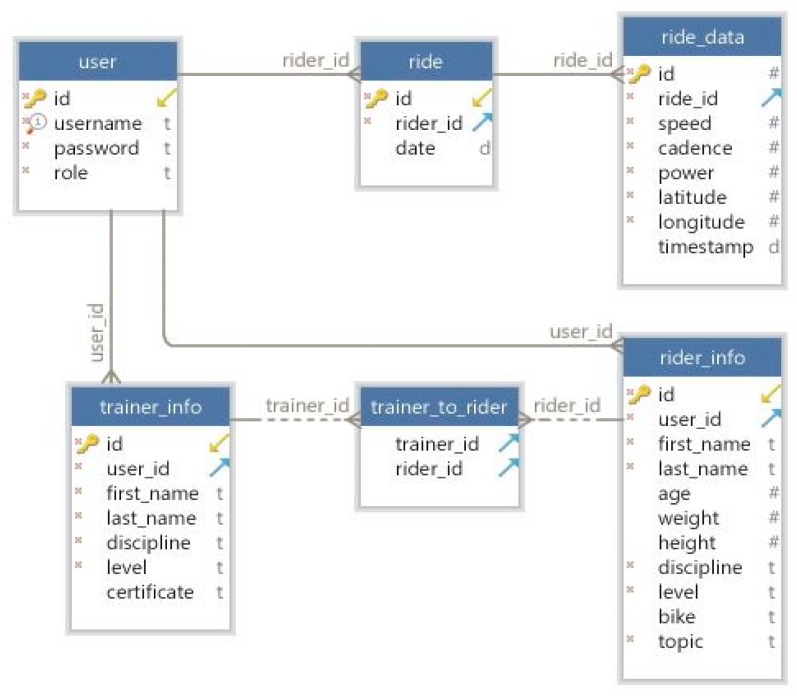
Database structure.

The database used is PostgreSQL where all the users’ data as well as the rides’ data are stored. The tables and the relationships can be seen in Figure 13.

The database consists of two tables that maintain records of all the rides for each cyclist (ride_table) and the data recorded for each ride (ride_data). General user data is kept specific to all users, regardless of profile, such as username, password, and role. Because users may have different roles, namely cyclist or trainer, it means that the specific data for each category will be different. Therefore, the rider_info table was created to keep specific information for cyclists such as name, weight, bicycle, and topic, the last one being used to send commands from the Android App to the rider’s bike frame module. The trainer_info keeps information about coaches such as name, discipline, level, and certificate.

Because a cyclist can have multiple trainers and a trainer can collaborate with multiple cyclists, there is a many-to-many relationship between the two entities. This was translated into a relationship table trainer_to_rider. This table keeps in each record the relation between a trainer and a cyclist through their primary keys.

The database was defined first by writing the tables and relationships as model classes in separate Python modules, all centralized in a package named models. After this, the database was created directly from the defined classes using the SQLAlchemy library with the Code-First approach. This library is an Object Relational Mapper (ORM) designed for Python that allows users to map database tables and relationships to classes and work with entities to perform CRUD operations (Create Read Update Delete). Everything from database access to creating queries are performed in the background, allowing the developer to write complex database operations that are translated into the database-specific queries.

The other Python package is named controller. Here, the Back-End is divided into two functionalities. The first one is dedicated to users. The UserView.py module defines all the methods that provide functionality for mobile and web applications using the table dedicated to users. These methods can create a new account, modify user data, and validate login credentials by querying the user, trainer_info, rider_info and trainer_to_rider tables with the help of SQLAlchemy. Similar to UserView.py, the RideView.py module contains all the methods dedicated to rides and rides data respectively, performing queries on the ride and ride_data tables. All the functions described above have a decorator that specifies the URL path and the type of REST (Representational state transfer) method through which they can be accessed remotely. Also, some methods may have more decorators depending on the access level. For example, the delete_ride method has both the *@Auth.auth_required* decorator, which allows it to be called only if the request has a token corresponding to an authenticated user, and the *@Auth.rider_required* decorator, which checks if the requester is a cyclist. These decorators are in fact other methods called implicitly to verify various requirements, such as whether the user is authenticated or has a certain role to access a resource. If the access criteria are satisfied, the decorated method will perform its task. All the decorators as well as the methods used to generate and decode JWT tokens are in the Authentication.py module. The encryption algorithm is SHA-256 and the Unicode standard is UTF-8.

In order for all the Back-End methods written in Python and described above to be remotely accessible over the Internet, a Web Server Gateway Interface (WSGI) is required. This mechanism handles all the HTTP requests coming from the Internet, routing them to the appropriate methods from the local machine. For this application, a WSGI server called Gunicorn was used, which is specifically designed to work with the Flask framework. It was configured to work as a daemon with three workers, so it did not interfere with the other running processes on the machine. These workers are actually threads in a pool, that are assigned per request to route to the corresponding method.

### 2.10. MQTT Broker

The communication between the MQTT clients was made possible by the Mosquitto broker, developed by Eclipse Foundation and released for public use for free. Once installed and configured, the broker starts as a background process when the Raspberry Pi finishes booting. The settings were configured in the mosquitto.conf file, enforcing the use of a username and password for all MQTT clients in this project for increased security. In addition, two listeners are configured here. The first uses the 1883 port for TCP/IP connection, which is used both for communication with the frame module and for sending commands and status information between the frame module and the Android Application. The second listener exposes the 8083 port for websockets connection, used by the Web Application for receiving online ride data from the frame module. Moreover, in this configuration file, the SSL certificates are also added for a more secure connection and the location where logs from Mosquitto broker will be stored.

### 2.11. Other Components

In addition to the Back-End and the MQTT broker, there are other auxiliary components that fulfill various roles on the Raspberry Pi server.

The first component is the Python service that subscribes to the data topic and retrieves ride data messages transmitted by the bicycle’s frame module and saves it to the database based on the ride id from the message. This service started at the Raspberry Pi’s boot time is waiting for the internet connection to be established, then it initializes the MQTT client and starts an infinite loop thread waiting for messages on the *connectedbike/data/#* topic. The hashtag character is a wildcard, meaning that all messages that are sent on the topic *connectedbike/data/{user_id}* will be received, regardless of the user id. This is advantageous as the ride_data table does not require a user id but only ride id, and this id is sent along with the data in the MQTT message.

**Figure 14:**
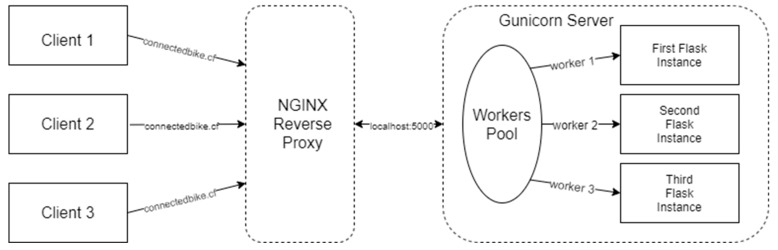
NGINX reverse proxy operation scheme.

Another important part of running the Back-End is the reverse proxy component. Its purpose is to hide implementation details and protect the Back-End component. All requests from clients are received by the reverse proxy that routes them to the WSGI and then routes back the response to the caller. For this project, NGINX was used as a reverse proxy, being a free and open-source software, supported by numerous contributors. After installation, it has been configured in the connectedbike.conf. Here were defined the access domains, the path to the Back-End, the files for storing logs, and the server that contains the parameters and the proxy and access control headers. Certbot certificates for SSL encryption are also configured here. Thus, all requests received on the public domain connectedbike.cf or www.connectedbike.cf are forwarded by the proxy to the Gunicorn WSGI serving the Back-End. The operation of this assembly can be seen in Figure 14.

After installation, the NGINX server automatically starts as a daemon service when the Raspberry Pi is turned on. Also, at the time of activation, NGINX takes over and sets all the configurations identified in the file with the .conf extension from the /etc/nginx/sites-available/ folder, in this case being connectedbike.conf.

The third server component is the Cron utility. This is a simple service offered on most Linux operating systems. To configure the crontab file, which the service uses as a look-up file for scheduling tasks, the *crontab -e* command line is called from the command line. For this application, a command is added to the crontab to run at boot time on the Raspberry Pi and to call the mqttdataposter.sh script. This shell script changes the current working directory to the root of the Application folder containing the MqttDataPoster.py. Afterwards, the Application is started from inside its Python virtual environment.

Both the MQTT Mosquitto broker and the NGINX reverse proxy server are automatically started at boot time and run as background services. Gunicorn, however, does not offer this possibility, which is why a forth auxiliary component is required, namely the creation of a Systemd unit with the .service extension, that handles the activation of the Gunicorn server at boot time. Systemd is a utility offered within the Debian operating system that allows the creation of user-defined services, running in the background. The service created for Gunicorn is called connectedbike.service and consists of three parts.

The first part, defined by the [Unit] tag, contains a description of the service and specifies the dependencies required to run the service. In this case, they are network-online.target and network.target.

The second part represented by the [Service] tag specifies the details for starting the service. Here the following are defined:

- *User = pi:* the Linux user running the service
- *Group = www*-*data*: the user group that runs the process
- *PIDFile = /tmp/gunicorn.pid*: the file that stores the process id
- Environment=”PATH=/home/pi/Programming/licentabackend/venv/bin” ”FLASK_ENV=production” ”DATABASE_URL=postgres://user:password@localhost:5432/db_licenta_deployment” ”JWT_SECRET_KEY=encodingstring”: the virtual environment for the Back-End and the environment variables
- *WorkingDirectory = /home/pi/Programming/licentabackend*: the folder in which the Flask application is located
- ExecStart={path_to_directory}/venv/bin/gunicorn –workers 3 – access-logfile {path_to_directory}/logs/gunicorn-access.log –error-logfile {path_to_directory}/logs/gunicorn-error.log -b localhost:5000 wsgi: the command to be executed when calling the service. This command calls the WSGI file that instantiates the Flask application, using Gunicorn and the virtual environment. The server will start running locally, serving port 5000 with three threads available for handling requests
- *ExecReload = /bin/kill -s HUP $MAINPID*: specifies how the service can be restarted
- *ExecStop = /bin/kill -s HUP $MAINPID*: specifies how the service can be terminated

The third section defined by the [Install] tag allows the service to be enabled or disabled. So, using the WantedBy=multi-user.target directive, a folder with the same name is created that persists even if the device is restarted and activates the service at boot time. In order for the service to be active and running every time the Raspberry Pi is started, the shell command *sudo systemctl enable connectedbike.service* is required. The same command, but replacing the enable keyword with start or stop, can be called to begin or terminate the service.

Last but not least, the Raspberry Pi server has allowed firewall access to the ports required by the application. Also, for a more efficient and user-friendly use, a free domain name (connectedbike.cf) has been assigned to the server’s IP.

### 2.12. Web Application

In this project, the web application has the role of displaying in a clear and easy way the data read by the module on the bicycle, giving the trainer the possibility to monitor the evolution of a cyclist online. In addition, the application offers the possibility of both categories of users to analyze previous rides. Also from the app, users can register a new account, login or connect with each other for collaboration. To achieve this, the ReactJS Javascript framework, developed and released free by Facebook, was used.

The application uses multiple components and elements to achieve the desired graphical user interface and user experience. For an easier state management, the react-redux library was used. It allows for centralizing the state for the entire application and passing only parts of it to components that specifically need them. The global application state can be modified only by functions named reducers. They receive as a parameter the current state of the application and the action based on which the decision to change the global state is taken.

For the implementation of this application, the tutorial presented by Jason Watmore in [26] is followed. The structure is divided into pages, auxiliary components and Redux components. In total, there are five pages: LoginPage, RegisterPage, RiderPage, TrainerPage and MonitoringPage. These in turn contain sub-pages, components rendered in the main page, which change according to the selected tab in the left-side menu. This allows conditional display of several different information, using the same URL. The user interface (buttons, labels, graphic elements) was created with the help of the MaterialUI library, which offers a wide range of free graphic elements. The monitoring page is accessible by both the trainer and the cyclist, being the place where the data is displayed, whether they come online or are uploaded from the Back-End. When the monitoring page is rendered in the browser, a props check is performed. If a ride id is found, then a function is called to bring the data through a GET request to the server. In the absence of a ride id, riderTopic is received in the props and the component subscribes to this topic to receive the data from the bicycle frame module in real time. To receive data through MQTT, the paho-mqtt library for React is used and the protocol supported by JavaScript is Websockets, which is why the broker on the server exposes both TCP/IP connection port and a Websocket connection port.

Because the data from the bicycle is transmitted and records each second, the number of entries in the database increases drastically. Thus, for the data to be fully displayed and analyzed, without occupying a large portion of the graphical interface, the Highcharts library for React was used. Three Highcharts charts were configured with this, one for speed, one for cadence and one for power. To each chart was attached a Navigator element available in the library that allows selection of a portion from a large data set and the graphical visualization of that part only, thus facilitating a thorough study of the ride in more detail. Also on the monitoring page was added a map using the GoogleMaps library for React, that displays markers with latitude and longitude received from the GPS module on the bicycle.

The components folder also includes AlertBar.js, which is used to display labels with various user information about in-app events such as failed login or failed connection to Back-End. Also here have been defined a set of constants for enforcing private routes, respectively limiting access to the application for users who have not logged in. So, anyone can access the Login or Register page, but only users who have logged in and received an authorization token from the server can navigate through the rest of the application. In addition, these constants decide based on the response from the server whether the user is a cyclist or coach and redirects it to the appropriate page.

Finally, yet importantly, the Redux part is composed of three parts:

1. Actions—here are the functions that can be called to change the overall state of the application. These have been divided into functionalities for user, rider, trainer, and alert. Within them, in addition to the implemented logic, is called the dispatch method in which a constant is sent as a parameter, which is respectively the part of the global state that must be modified;
2. Constants—here are defined the constants based on which the behavior of the global state is decided. Similar to actions, constants are also divided into functionalities for user, trainer, rider, and alert;
3. Reducers—represent the functions that perform the actual change of the global state. They are called with the initial state or the action as parameters. Based on one of the constants in point two, which is transmitted in the body of the action, the state is modified with the new value sent in the body of the action;

The engine behind these three parts is the store. It is created using the createStore function in the redux library. This function is called with a set of parameters such as persistedReducer or middleware. In order to maintain the entire state of the application, even after the user refreshes the browser page, the redux-persist library is used. With it, the entire status hierarchy of the application is saved in Local Storage as JSON, where it can be retrieved again after refreshing the web page.

In the application development stage, the Node.js server was used. It has the role of running a local instance (localhost) at a predefined port, in this case, port 3000, where the developer can observe the changes made to the code. To expose the application on the internet and for anyone to access it, the npm run build command was run, creating a new build folder containing all the files and packages needed to run the application in production. This build was uploaded for free to Netlify, making it accessible to everyone on the internet.

## 3. Results

The main distinguishing features of the system are presented in the following:

- The architecture of the project allows the system to be divided into dedicated modules, each implementing both data computing and transmission operations. This approach offers the advantage of reducing computational demand on a single module by distributing specific operations such as conversions or scaling to each module separately;
- By using a real-time operating system on the Arduino Mega from the bicycle frame module, a clear definition of the tasks and their separation according to the fixed duration are obtained;
- The data transmission between the pedal module to the frame module is done via infrared communication, overcoming the wires limitation imposed by the two rotational couplings from pedal to bike frame;
- In order to use a single three-wire load cell, it was necessary to close the Wheatstone bridge with the resistors of equal value and minimum tolerances;
- The Raspberry Pi was turned into a control component capable of stopping or starting recording ride activity depending on the commands received from the Android App;
- The MQTT Protocol was used as the main communication medium in the system, being used both for transmitting on-line data collected from sensors and for full-duplex communication between the bicycle module and the mobile phone. This reduced the complexity of the transmission and increased the robustness of the whole assembly;
- Multiple security levels were implemented such as password encryption with secret key, JWT authorization tokens for Back-End, restricting access to MQTT channels by username and password and implementing SSL certificates on the server for both Back-End and MQTT broker;
- The Redux library was used to manage the entire state of the application and its persistence in the local memory of the browser so that it is not reset between refreshes.

**Figure 15:**
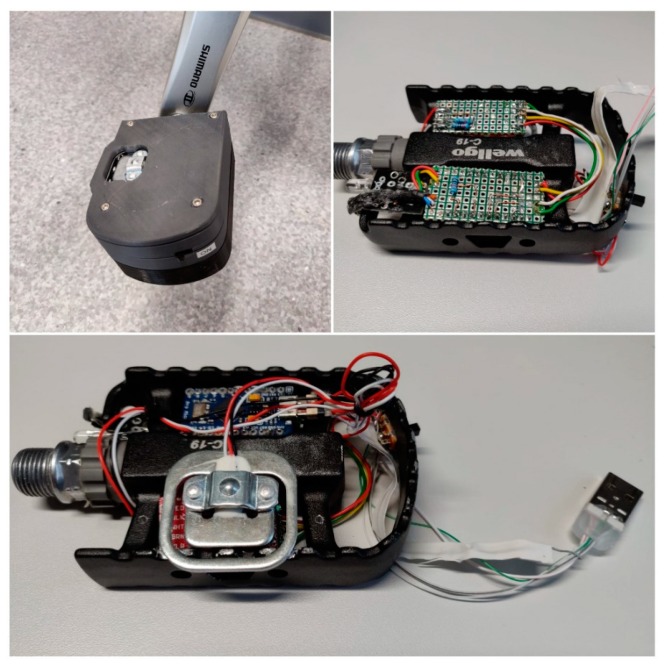
The final pedal module.

The pedal module, as mounted on the bicycle, can be seen in Figure 15.

**Figure 16:**
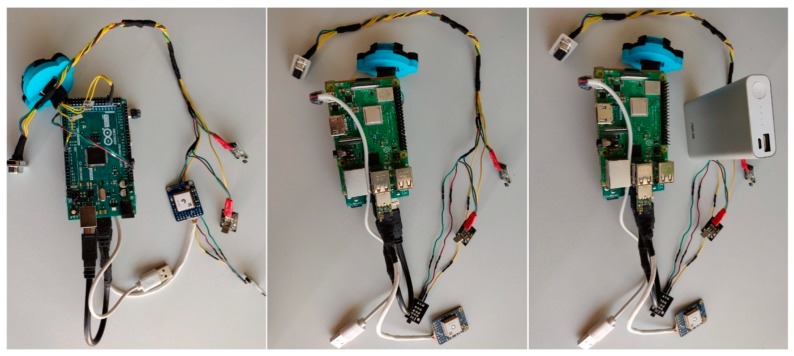
The resulted bicycle frame module.

Also, the frame module, consisting of Arduino Mega 2560, Raspberry Pi 3 Model B+, Hall sensors, infrared receiver, and GPS module from Adafruit is shown in Figure 16.

**Figure 17:**
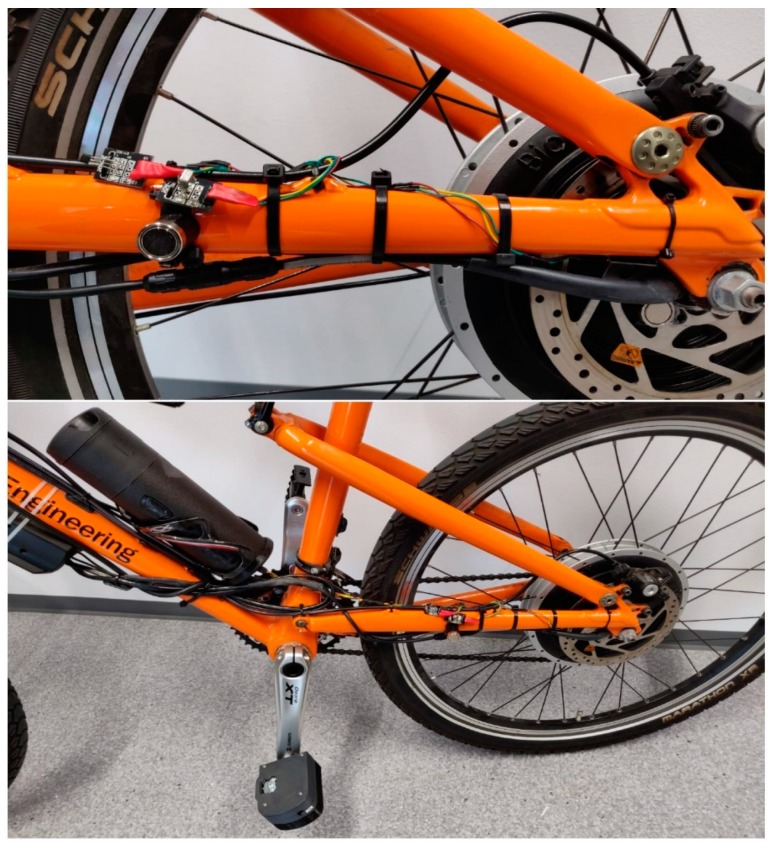
Final form of the Connected Bike

In its final form, the hardware part of the frame module has been placed inside a bicycle water bottle and the connection with the sensors is made through an RS-232 connector as shown in Figure 17. This allows the bottle to be disconnected from the electronic components on the frame, leaving the sensors in place.

**Figure 18:**
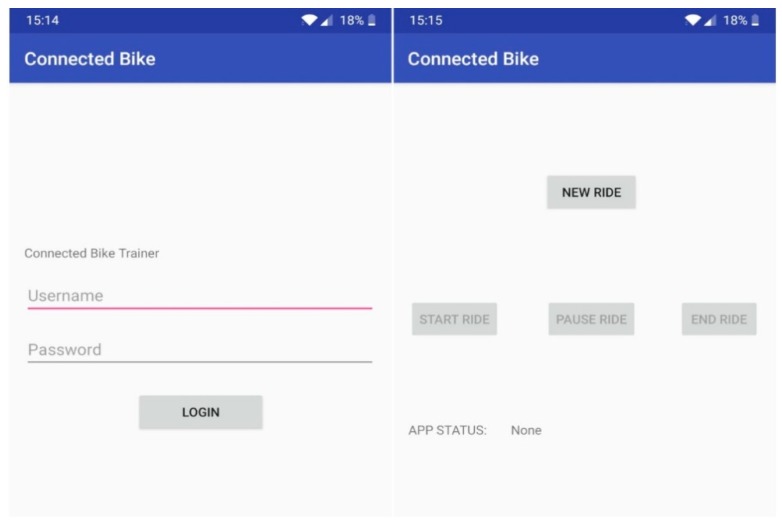
Android application interface

The Android App that allows the cyclist to manage the ride data monitoring is presented in Figure 18.

**Figure 19:**
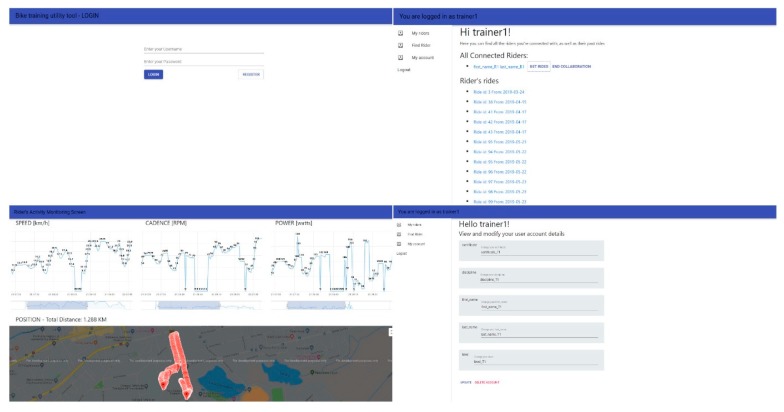
Web application of the Connected Bike

Last but not least, the web application by which users can create their account, monitor the rides or create connections with each other is shown in Figure 19.

Finally, using the above modules, both hardware and software, the following requirements were achieved:

- Collecting raw data from the two Hall sensors and from the load cell
- Computing the riding speed [km/h] with a precision of two decimals, pedal cadence [rpm] and instantaneous power from the cyclist [watt]
- Track the cyclist’s position in real-time by reading latitude and longitude from the GPS module
- Sending with a 1 s period the collected and processed data from sensors to the server to be stored in the database and for online monitoring
- Implementing an Android Application that allows the rider to create a new ride, start or terminate the current one, and also receive information on the state of the bike’s frame module
- Creating a Web Application through which the data sent from the bicycle can be viewed both in real time and after the ride has been completed
- Securing the application by working with sessions, authorization tokens, SSL certificates, and credentials for the MQTT clients

## 4. Conclusions

This connected bike project was born out of a real issue encountered among both amateur and professional cyclists. This problem consists of the limitations existing in the current training methods. In this project, a wide range of technologies have been used, starting from the electronics and hardware side and to the web and mobile applications. Putting all these together, a prototype of a complete solution has been created to help cyclists and facilitate trainers.

## Abbreviations

AUTOSAR	Automotive Open System Architecture
CRUD	Create Read Update Delete
GPIO	General-Purpose Input/Output
GPS	Global Positioning System
HTTP	Hypertext Transfer Protocol
ICR	Interrupt Capture Register
IoT	Internet of things
IP	Internet Protocol
IR	Infrared
JSON	JavaScript Object Notation
JWT	JSON Web Token
LED	Light-Emitting Diode
MIT	Massachusetts Institute of Technology
MQTT	Message Queues Telemetry Transport
MVC	Model-View-Controller
NGINX	engine X
ORM	Object Relational Mapper
RST	Representational state transfer
RTE	Runtime Environment
RTOS	Real Time Operating System
SEMS	smart e-bike monitoring system
TCNT	Timer Counter Register
TCP	Transmission Control Protocol
UART	Universal Asynchronous Receiver-Transmitter
UML	Unified Modeling Language
URL	Uniform Resource Locator
USB	Universal Serial Bus
WSGI	Web Server Gateway Interface
